# Surfactin as a Green Agent Controlling the Growth of Porous Calcite Microstructures

**DOI:** 10.3390/ijms21155526

**Published:** 2020-08-01

**Authors:** Anna Bastrzyk, Marta Fiedot-Toboła, Halina Maniak, Izabela Polowczyk, Grażyna Płaza

**Affiliations:** 1 Department of Process Engineering and Technology of Polymer and Carbon Materials, Faculty of Chemistry, Wrocław University of Science and Technology, Wybrzeże Wyspiańskiego 27, 50-370 Wrocław, Poland; izabela.polowczyk@pwr.edu.pl; 2 Łukasiewicz Research Network-PORT Polish Center for Technology Development, Stabłowicka 147, 54-066 Wrocław, Poland; 3 Department of Micro, Nano and Bioprocess Engineering, Faculty of Chemistry, Wrocław University of Science and Technology, Wybrzeże Wyspiańskiego 27, 50-370 Wrocław, Poland; halina.maniak@pwr.edu.pl; 4 Department of Environmental Microbiology, Institute for Ecology of Industrial Areas, Kossutha 6, 40-844 Katowice, Poland; g.plaza@ietu.pl

**Keywords:** biomineralization, calcite, vaterite, biosurfactant, Turbiscan, TGA, XRD

## Abstract

This study presents a new, simple way to obtain mesoporous calcite structures via a green method using an eco-friendly surface-active compound, surfactin, as a controlling agent. The effects of synthesis time and surfactin concentration were investigated. The obtained structures were characterized by X-ray powder diffraction (XRD), scanning electron microscopy (SEM), thermogravimetric analysis (TGA), and differential scanning calorimetry (DSC) coupled with gas mass spectrometry (QMS) analysis. The experimental data showed that surfactin molecules significantly changed the morphology of the calcite crystals, roughening and deforming the surface and creating a greater specific surface area, even at low biosurfactant concentrations (10 ppm). The size of the crystals was reduced, and the zeta potential value of calcium carbonate was more negative when more biosurfactant was added. The XRD data revealed that the biomolecules were incorporated into the crystals and slowed the transformation of vaterite into calcite. It has been shown that as long as vaterite is present in the medium, the calcite surface will be less deformed. The strong influence of surfactin molecules on the crystal growth of calcium carbonate was due to the interaction of surfactin molecules with free calcium ions in the solution as well as the biomolecules adsorption at the formed crystal surface. The role of micelles in crystal growth was examined, and the mechanism of mesoporous calcium carbonate formation was presented.

## 1. Introduction

Calcium carbonate (CaCO_3_) is a common biomaterial produced by living organisms for building shells and exoskeletons such as seashells, avian eggshells, and bones to support and protect their bodies [1]. This biomaterial possesses unique properties (high surface area, high porosity, high mechanical strength, and non-toxicity) that can offer wide potential applications in industry as filler materials for paints, pigments, coatings, paper and plastics, carriers of active compounds and nanoparticles for drug delivery, and matrices for polymeric capsules [2,3,4,5,6,7]. CaCO_3_ occurs in different crystalline polymorphs: anhydrous phases of aragonite, vaterite and calcite, and hydrated phases of calcium carbonate monohydrate, calcium carbonate hexahydrate and amorphous calcium carbonate [8]. Each of these crystalline forms is characterized by various morphologies and physicochemical properties. For instance, spherical hexagonal vaterite exhibits higher porosity and can decompose rapidly under relatively mild conditions compared with rhombohedral calcite [2]. Aragonite, on the other hand, forms thin needles and has a strong tendency to form spherulitic clusters with high porosity and density [9]. In biology, biomineralization can occur at specific sites on the cell wall, in spaces between closely packed cells, inside enclosed compartments within a cell or in spaces outside a cell. The specific mineralization sites can include phospholipid vesicles, protein vesicles, cellular assemblies, and molecular frameworks [10]. The formation of crystalline polymorphs with desired properties is controlled by different and occasionally synergic processes, such as the regulation of respective ion concentrations, the formation of initial amorphous and other metastable precursor phases, and the presence of specific biomolecular additives [11]. Extensive research has shown that living organisms require biomolecular additives such as proteins and polysaccharides with carboxyl, phosphate and sulfate groups to control the hierarchical structure and the mechanical properties of calcium carbonate [10,12]. These biomolecules can act as templates and control the polymorphs, orientation and morphology of calcium carbonate. Thus, research has been focused for many years on learning and mimicking the biosynthesis of calcium carbonate. The ability to control the particle shape, size and morphology is fundamental from the viewpoint of technical applications [13]. Therefore, finding a way to obtain calcium carbonate with specific properties remains a challenge, especially with the use of “green additives”. These “green compounds” include biosurfactants, the surface-active molecules produced by living cells. Compared to synthetic surfactants, biosurfactants are non-toxic, easily biodegradable and can be synthesized from renewable waste with an appropriate ratio of carbohydrates and lipids to support optimal bacterial growth [14].

Surfactin (C_53_H_93_N_7_O_13_) is an anionic lipopeptide biosurfactant with two carboxyl groups that has been widely used in the “green synthesis” of silver and gold nanoparticles [15]. Our previous research has shown that surfactin can also be used effectively as a calcium carbonate growth controlling agent. CaCO_3_ structures obtained in the presence of surfactin were characterized by large irregularities (terraces and depressions) on the surface of the crystals during a short ageing period [16]. To better understand the mechanism of crystal growth, this study investigated how surfactin concentration can affect the morphology, polymorphs and properties of calcium carbonate precipitated in an aqueous solution after prolonged ageing. The kinetics of crystal growth in the presence of surfactin molecules were also examined. As a result, we identified a new role of surfactin in the biosynthesis of porous calcite. The porous calcite formed and modified by biosurfactants can offer many new possibilities in biomedical and environmental applications.

## 2. Results and Discussion

**Figure 1:**
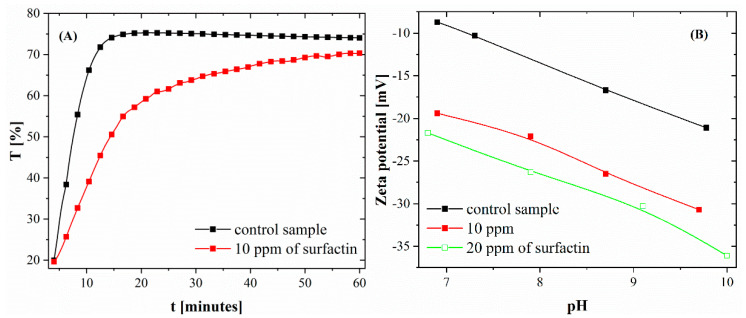
(**A**) T (%) value changes during the time of reaction. (**B**) Zeta potential of calcium carbonate precipitated at various surfactin concentrations after 24 h of ageing (NaCl concentration was 1 mM, standard deviation for zeta potential values was lower than 1 mV).

The calcium carbonate particles were synthesized in a spontaneous, rapid reaction in which calcium chloride and sodium carbonate were used as sources of calcium and carbonate ions, respectively. In the control test (without biosurfactant), turbidity appeared immediately after the Na_2_CO_3_ solution (pH 11.3) was poured into the CaCl_2_ solution without biosurfactant (pH 8.0), indicating the precipitation of CaCO_3_ particles. After 5 min of reaction, the sample vial was inserted into the measuring cell of the Turbiscan apparatus, and changes in the value of light passing through and reflected from the sample were recorded. Over time, the light transmittance value (*T*) increased significantly from 20 to approximately 75, reaching a plateau after approximately 15 min (Figure 1A). A high value of *T* (%) indicates very unstable systems [17], in this case due to the formation of larger calcium carbonate particles that sediment. Similar results were observed where Ca^2+^ ions were in contact with biosurfactant particles for 24 h. However, in the samples with surfactin, the *T* (%) value increased much more slowly, reaching a value of approximately 70 after 1 h (see Figure 1A). This is the result of the interaction of biomolecules during the process (nucleation and further growth of particles).

To better understand the process, the ageing time was extended to 24 h, different surfactin concentrations were applied, and the polymorphic form of the crystals and morphology of the structures were analysed. Physicochemical and thermal properties of the biomineral were also measured.

### 2.1. Particle Size Distribution and Zeta Potential of Calcium Carbonate after 24 H of Ageing

The particle size distributions and the diameters of CaCO_3_ particles obtained in the control system and with surfactin after 24 h of ageing are shown in Figure S1 and Table 1, respectively. The addition of surfactin to the reaction system resulted in a significant particle size reduction. The median diameter (*d_50_*) of CaCO_3_ formed without the biosurfactant was 24.4 μm. Increasing the concentration of surfactin molecules up to 20 ppm decreased the value of *d_50_* to 10.6 μm, and more particles with diameters lower than 5 μm were formed. Comparing the data obtained with 10 and 20 ppm surfactin, one can see that the difference in the size of the resulting particles was not significant. This means that surfactin effectively blocked the growth of CaCO_3_ crystals, even at 10 ppm.

The presence of surfactin molecules in the reaction system changes not only the size but also the zeta potential value of CaCO_3_ particles. Based on the data presented in Figure 1B, it can be seen that the zeta potential value of particles in the control sample changed from −8.1 to −21.1 mV in a pH range from 7 to 9.8. The addition of surfactin at concentrations of 10 and 20 ppm increased the negative value of the zeta potential to −30 and −36.1 mV, respectively, at a pH of approximately 10 ± 0.3. The changes in zeta potential value were also observed by other researchers using polypeptides, fulvic acid or stearic acid [18,19]. From previous data in the literature, it is known that biomolecules can adsorb on solid faces formed as a result of the crystallization process and change the interfacial energy [20,21]. This may explain the decrease in particle size in the presence of surfactin, as shown in Figure S1 and Table 1. The high negative value of zeta potential of crystals obtained in the presence of surfactin proves that biosurfactant particles are present on the surface of particles.

### 2.2. XRD Analysis of Calcium Carbonate after 24 H of Ageing

**Figure 2:**
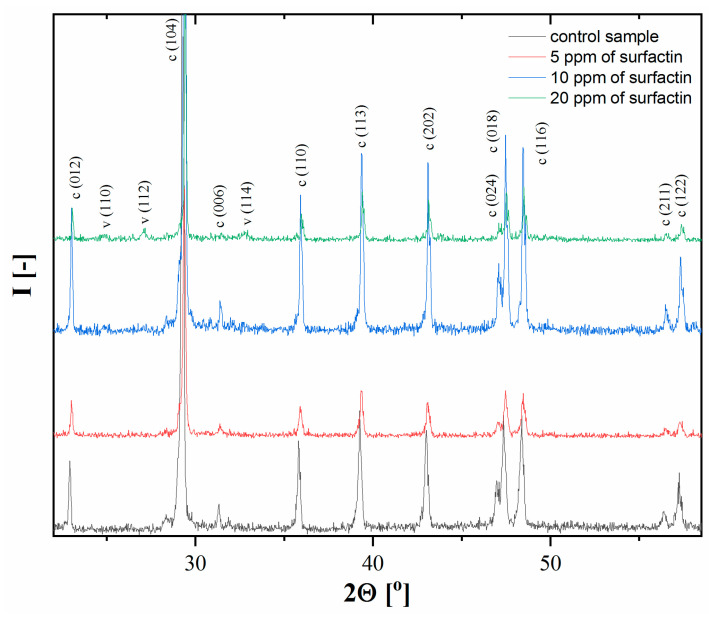
XRD patterns of calcium carbonate precipitated at different surfactin concentrations after 24 h of ageing. The concentration of CaCl_2_ and Na_2_CO_3_ was 5 mM (c-calcite, v-vaterite).

Crystalline calcium carbonate polymorphs obtained after 24 h of ageing were determined by XRD analysis. From Figure 2, it can be seen that in the control sample (without surfactin) and in the presence of 5 ppm surfactin, only calcite crystals were obtained. This means the complete transformation of the metastable phase, vaterite, into a thermodynamically stable phase, calcite, under these conditions. A further increase in surfactin concentration up to 20 ppm resulted in a mixture of calcite and vaterite particles, which was revealed by the characteristic peaks in Figure 2.

For 10 and 20 ppm surfactin in the reaction system, the vaterite content was found to be 1.4% and 14.9%, respectively (Table 2). This means that the higher concentrations of surfactin molecules can effectively delay the transformation of vaterite to calcite. In all investigated samples, a trace amount of Na_2_CO_3_ (0.1%, at 2Ө = 28°) was found. On the basis of the calculated unit cell parameters of the polymorphs (Table 2), a possible incorporation of surfactin into the crystal structure of calcite was found. For that purpose, the relative changes (Δ*l*, Δ*V*, %) in the lengths of a and c edges and volume in the elemental cell for pure calcite without surfactin (*l_0_*, *V_0_*) and for systems with varied amounts of surfactin (*l_s_*, *V_s_*) were calculated: Δ*l* = (*l_s_* − *l_0_*)/*l_0_*·100. In all cases studied, a slight elongation of 0.046–0.054% and 0–0.04% for the a and c edges of the calcite elemental cell, respectively, was observed. Nevertheless, the increase in the edge dimensions did not correspond directly to an increment of surfactin concentration added to the reaction medium and seemed to become rather independent of its higher values (10 and 20 ppm). This small elongation of the edges, however, influences the volume of the calcite elemental cell (Table 2). For the subsequent concentrations of 5, 10 and 20 ppm surfactin, the calculated relative changes in the volume of calcite were 0.085, 0.142 and 0.144%, respectively. Similar results were observed for biogenic calcite and for calcite obtained in the presence of amino acids and proteins [22,23]. The changes in the elemental cell parameters seem to indicate that surfactin may induce anisotropic positive lattice distortion due to the possible incorporation of biomolecules into the crystal lattice. This phenomenon may influence the crystal structure of calcite in all studied systems. The effect of surfactin on the changes in the edge length and volume of the vaterite elemental cell is difficult to determine, as Table 2 shows, the increase in the values of particular parameters is in the range of standard deviation.

### 2.3. Morphology of Calcium Carbonate Crystals after 24 H of Ageing

**Figure 3:**
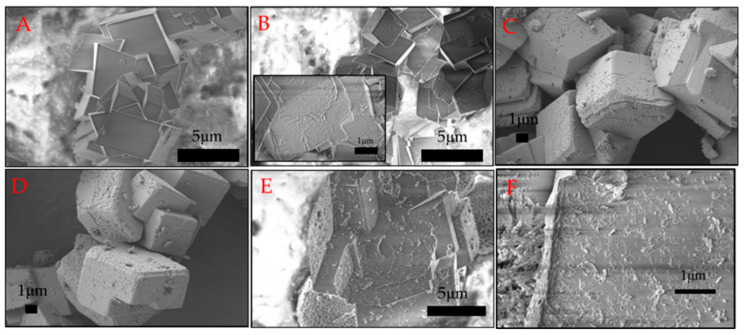
SEM images of calcium carbonate precipitation at various surfactin concentrations after 24 h of ageing. (**A**) control sample, (**B**) 5 ppm, (**C**,**D**) 10 ppm, (**E**,**F**) 20 ppm of surfactin. The concentration of CaCl_2_ and Na_2_CO_3_ was 5mM.

The morphology of the obtained calcium carbonate crystals was analysed using scanning electron microscopy, and the images are presented in Figure 3. In Figure 3A, it can be seen that without biomolecules, rhombohedral structures with a smooth surface and sharp edges characteristic of calcite were formed. The morphology changed when the biosurfactant was added to the reaction medium (Figure 3B–F). The surface of the crystal formed in the presence of 5 ppm surfactin was deformed as some irregularities appeared (Figure 3B). By analysing the images in Figure 3, with higher biosurfactant concentrations, the presence of spherical structures characteristic of vaterite was observed, which was also confirmed by XRD analysis (Figure 2). However, the most interesting morphology was observed for calcite. Increased surfactin concentration resulted in a greater surface roughness of the calcite crystals, with spherical and oval cavities on the surface. Moreover, the edges of the crystals were not so sharp and were significantly deformed (Figure 3C–F). The deformation of calcite crystal microstructures after adding compounds of biological origin has been widely described in the literature [24,25,26,27,28]. Biomolecules, especially anionic molecules, can bind calcium ions in solution as well as on the surface of created crystals, changing the direction of growth or blocking the growth of the crystal surface [2]. Surfactin is a surfactant that reduces the surface tension of water to 28 mNm_−1_ at a critical micellization concentration (CMC) in the range of 10–20 ppm, depending on pH and ionic strength [29]. Our previous studies showed that in a 5 mM aqueous solution of CaCl_2_ at pH 8, the CMC value for surfactin was 4.6 ppm [16]. Thus, the porosity of the calcite surface was a result of the formation of biosurfactant micelles in the reaction medium at surfactin concentrations above the CMC value. Similar results were obtained in the presence of nanosized spherules and worms of polymers and casein micelles [27,30].

In our studies, it was noted that more micelles present in the system lead to more porous calcite crystals. Comparing these results with previous results [16], it was observed that by extending the reaction time to 24 h, porous crystals are formed at twice the biosurfactant concentration. These structures may have great potential for use as inorganic matrices or carriers of active compounds or nanoparticles. It can be assumed that micelle concentration and ageing time play a crucial role in the formation of porous calcite structures.

### 2.4. BET Analysis of Calcium Carbonate after 24 H of Ageing

One of the most important parameters describing inorganic matrices is the value of specific surface area (SSA) as well as the size and volume of pores. The hysteresis loops observed in Figure S2 indicate the mesoporous structure of CaCO_3_. It was observed that particles obtained without biomolecules had a small SSA value of 0.18 m^2^g^−1^ (Table 3). This is the result of rhombohedral calcite formation with a smooth surface, as shown in Figure 3A. The increase in surfactin concentration to 10 ppm and 20 ppm significantly increased the value of SSA to 1.67 and 4.87 m^2^g^−1^, respectively. A higher value of the SSA arises from the roughness of the calcite surface formed in the presence of biosurfactant (Figure 3E,F). A larger specific surface area was obtained when a higher concentration of biosurfactant was added to the reaction mixture. From the data in Table 3, it can be seen that the average pore diameter increased from 8.7 to 11.7 nm when surfactin at higher concentrations was added. Moreover, the average pore volume doubled with increasing biosurfactant concentration. This may be due to the formation of more micelles in the solution as well as the fact that at a 20 ppm concentration of biosurfactant, more vaterite was formed (Table 2). It is known from the literature that vaterite has a higher SSA than calcite [31]. 

Comparing these data with our previous results [16], one can say that after 24 h of ageing, the specific surface area and average pore volume slightly decreased from 7.11 to 4.87 m^2^g^−1^ and from 0.0202 to 0.0123 cm^3^g^−1^, respectively. This was related to the reduction of vaterite content and the incorporation of more biosurfactant molecules into calcite crystals.

### 2.5. Thermogravimetric Characteristics of Calcium Carbonate Structures

**Figure 4:**
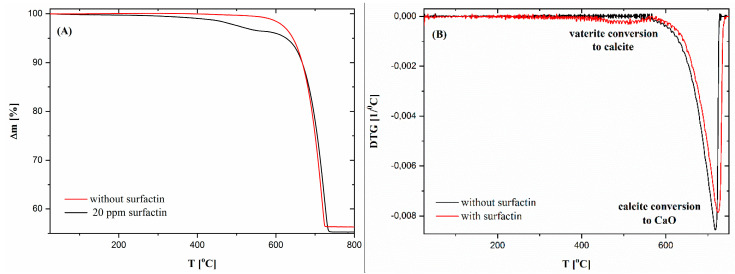
TG and DTG curves of the calcium carbonate structures obtained without and with 20 ppm of biosurfactant. Time of reaction was 24 h. The concentration of CaCl_2_ and Na_2_CO_3_ was 5 mM. (**A**) TG; (**B**) DTG.

Thermogravimetric analysis of calcium carbonate samples with 5 Kmin^−1^ heating rate was also performed. To show the effect of surfactin on the thermal behaviour of CaCO_3_, samples with the most differing surfactin content were selected, namely, without and with 20 ppm surfactin. It was observed that in both samples, with and without biosurfactant, the main degradation process started above 690°C, which was related to the conversion of calcite into calcium oxide (Figure 4A). Despite the fact that two additional weight losses were detected at lower temperatures (Figure 4A). The first loss had no specified maximum on the DTG curve. The second loss was observed only for the sample with surfactin and had a broad DTG peak with maximum about 510 °C (Figure 4B). From the literature, it is known that the first weight loss is related to the evaporation of moisture contained in the samples, and the second weight loss is related to the evaporation of water bound in the CaCO_3_ structure and/or transformation of vaterite to calcite [32,33]. To compare the chemical composition of the sample with and without surfactin, the weight loss observed during this two recesses was determined. The temperature ranges was selected according to DTG results. It was shown that in the first range (25–400 °C), the weight of the material with surfactin decreased by 0.95%. Continued heating resulted in a further decrease in material weight to the same value. For a sample without surfactin, it was not possible to divide this temperature region for two processes, as any DTG maximum in this area was observed. For this reason, the weight loss of this material in one temperature range from 25 to 566 °C was measured. Its value was approximately 0.71%. The higher weight loss value in this temperature range (25–566 °C) for the sample with surfactin could be connected with better moisture adsorption and surfactin molecule degradation. Also, these differences were visible in the total weight loss of the samples, which was higher for the sample with surfactin by approximately 1%. The maximum DTG associated with calcite conversion was also higher for this sample with surfactin, which could demonstrate obstruction of this process by the use of biosurfactant (Figure 4 and Table 4). 

**Figure 5:**
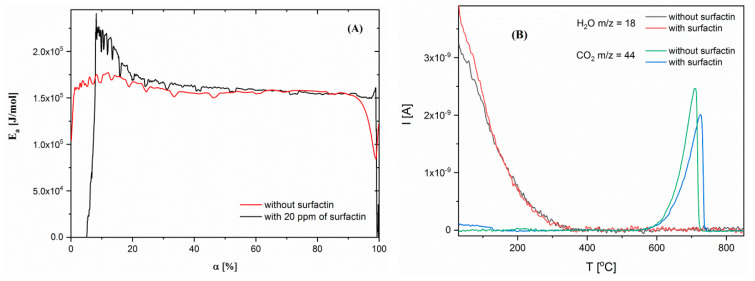
(**A**) *E_a_* changes as a function of materials conversion. (**B**) QMID (Quasi Multiple Ion Detection) value for m/z 18 (water) and 44 (CO_2_).

In addition, model-free kinetics were used to determine activation energy values as a function of material conversion [34]. To achieve this, both samples were analysed by the thermogravimetric method with four different heating rates (5, 10, 15, and 20 Kmin^−1^) and presented as a conversion curve (Figure S3). The activation energy value was calculated as follows (1):

(1) $$\frac{d\alpha }{dt}=Aexp\left(\frac{-E_{a}}{RT}\right)f\left(\alpha \right)$$

where, *α* is conversion, *t*—time, *A*—frequency factor, *R*—gas constant, *E_a_*—activation energy, *T—*temperature. A comparison of the obtained results for samples without and with 20 ppm surfactin showed that they differed from each other. For a material without surfactin, the *E_a_* value was almost constant over the entire conversion range (~160 kJmol^−1^). This means that only one process was observed during thermal degradation of the control sample—calcite conversion to calcium oxide. While analysing the sample with surfactin, it was observed that the first *E_a_* value was very low at the beginning of the experiment. Then, it increased rapidly to 250 kJmol^−1^. Further activation energy decreased to a similar value as for the sample without surfactin. Three processes can be defined during the thermal degradation of this sample. The first was assumed to be moisture release, the second was the conversion of vaterite to calcite or bonded water evaporation, and the third was the decomposition of calcite to CaO (Figure 5A). 

During the TG results interpretation, the thesis was put forward that the first degradation process was related to water evaporation and the second one to bound water evaporation and/or vaterite transformation to calcite. To verify this mass spectrometry, analysis was performed. In QMS studies, changes in ionic current as a function of temperature were investigated for specific m/z values for water (18) and carbon dioxide (44). The obtained curves demonstrated that in both samples (with and without surfactin), water was released during the first thermal degradation (till about 400 °C). These results excluded the thesis about the evaporation of bound water from the samples at approximately 510 °C (Figure 5B). For this reason, it was concluded that this process is most likely related to the transformation of vaterite to calcite. Such broad temperatures range for water release from the samples could indicate that their molecules are not only physically but also chemically adsorbed on the material surface, for which desorption higher temperatures are needed [35,36]. The changes in m/z 44 related to CO_2_ confirmed that this gas is evolving during the thermal degradation of CaCO_3_. It can also be seen that this process occurs in a higher temperature range for the sample with surfactin (Figure 5B). This is consistent with the results of the TG analysis (Figure 4 and Figure 5A). Additionally, another important conclusion can be drawn from these studies. It has been noted that at temperatures below 200 °C, CO_2_ was derived from the sample with surfactin, which was not indicated for the reference sample. This confirms the incorporation or adsorption of surfactin on the CaCO_3_ surface (Figure 5B). All observations were consistent with the XRD data (Table 2) and SEM images (Figure 3), which revealed vaterite in the sample precipitated with a surfactin concentration of 20 ppm.

### 2.6. Effect of Ageing Time on Crystal Growth

**Figure 6:**
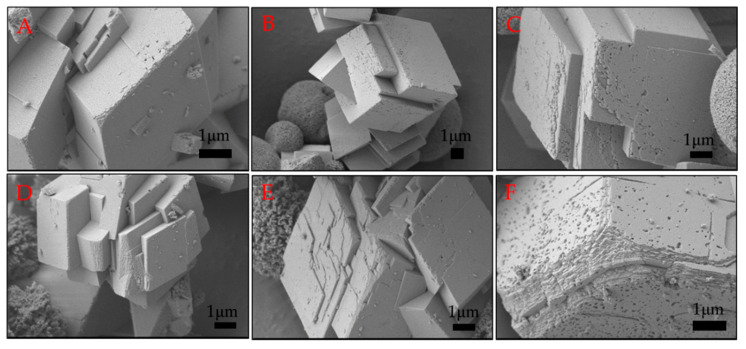
SEM images of calcium carbonate precipitated in 10 ppm of surfactin. (**A–C**) 30 min of aging, (**D**) 1 h of aging, (**E**) 3 h of aging, (**F**) 24 h of aging. The concentration of CaCl_2_ and Na_2_CO_3_ was 5 mM.

To describe more in detail how the porous structures of calcium carbonate were formed, as shown in Figure 3, the effect of ageing time on the crystalline structure and morphology of crystals was studied. The experiments were carried out in a reaction system containing 10 ppm surfactin. It was observed that in the system without the biosurfactant, pure calcite was precipitated in all studied processes during the time periods (Figure S4A). The presence of biosurfactant molecules led to the formation of a mixture of calcite and vaterite even after 24 h (Figure S4B). The mass fraction of the resulting phases is given in Table 5. As expected, the amount of vaterite decreased with extending reaction time. The highest concentration of vaterite was 40.5% after 30 min of ageing, and the lowest (1.4%) was after 24 h of ageing. As shown in the control sample, pure calcite was formed just after 30 min of ageing (Figure S4A). Therefore, this proves that biosurfactant molecules slow down the conversion of vaterite into calcite. When analysing the SEM images (Figure 6A–F), it was noted that more surface irregularities appeared during longer reaction times. More cavities were visible, and the edges of calcite were significantly deformed, showing a multilayered structure after 24 h of ageing. Thirty minutes of ageing led to the formation of calcite with slightly deformed edges. Few cavities were visible on the calcite surface. This may be due to the large amount of vaterite that was formed in this process, which was 40.5% (Table 5).

**Figure 7:**
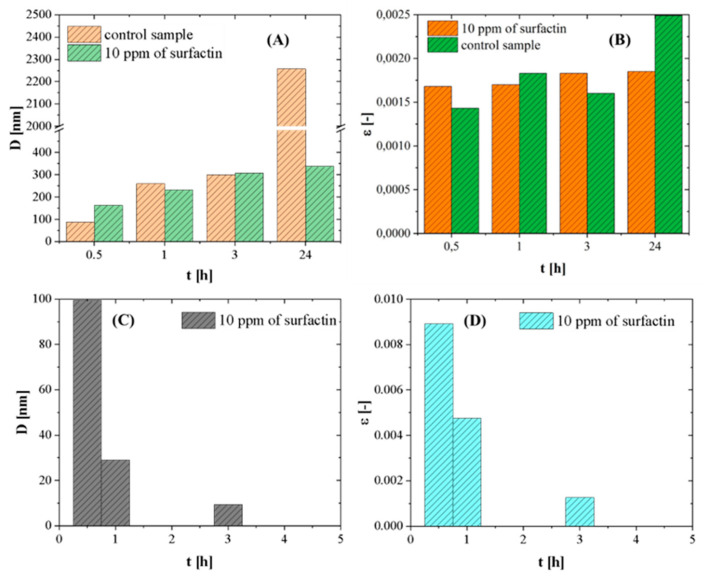
Changes in crystallite size (*D*) and microstrains (*ε*) of CaCO_3_ crystals obtained with and without surfactin. Calcite (**A**,**B**); vaterite (**C**,**D**).

Additionally, the changes in the average crystallite size (*D*) and microstrain (*ε*) of vaterite and calcite in samples as a function of time were calculated according to the Williamson-Hall method (W-H method) [37]. It was observed that the size of calcite crystallite in both samples increased with the progress of the reaction. The values of *D* and *ε* in the control sample were higher than those in the sample with surfactin. The crystallite size in the control sample increased sharply after 24 h (Figure 7A), which is characteristic for the crystallization process. By analysing the microstrain results, it was observed that their values were at similar levels for both samples. The only change was noticed in the control sample after 24 h (Figure 7B). This shows that surfactin molecules influenced the crystallization process of calcium carbonate. An increase in the size of crystallites and microstrains of calcite results from the clasical ion by ion attachment as well as the oriented aggregation. The increase in the microstrain is due to formation of crystal-crystal interface [38]. The lower value of *D* and *ε* after 24 h results from the incorporation of organic additivities into the crystals as well as decreasing the crystals boundary density [39]. 

Crystallites of vaterite were found only in the sample with surfactin. Their size and microstrain values decreased as a function of time due to dissolution/recrystallization reactions (Figure 7C,D). 

To understand the observed phenomenon, the mechanism of CaCO_3_ crystallization in the presence of surfactin should be analysed. According to the literature, the mixing of Ca^2+^ with CO_3_^2-^ ions results in the formation of nuclei and the synthesis of amorphous calcium carbonate (ACC) nanoparticles [28]. The precursor phase is highly hydrated and the most unstable phase of calcium carbonate. The ACC nanoparticles are equilibrated in solution quickly (within seconds) and transformed into a calcium carbonate polymorph (vaterite). The transformation of ACC to vaterite can occur through a direct solid-state transformation, and it is experimentally difficult to capture [40]. ACC nanoparticles aggregate into larger particles, and dehydration of the precursor phase takes place. This leads to the formation of spherical vaterite particles, a metastable phase. Vaterite is quickly combined in surface-mediated dissolution-reprecipitation reactions, leading to the formation of pure rhombohedral calcite. This leads to a reduction in the number of crystals and an increase in particle diameter, as shown in Figure 1A.

Based on the obtained results and literature data, it can be assumed that surfactin molecules act in the biomineralization of calcium carbonate at two different stages, which are mutually dependent:

(a) in solution during the nucleation stage (formation of Ca-surfactin complexes),
(b) on the surface of formed CaCO_3_ crystallites.

**Figure 8:**
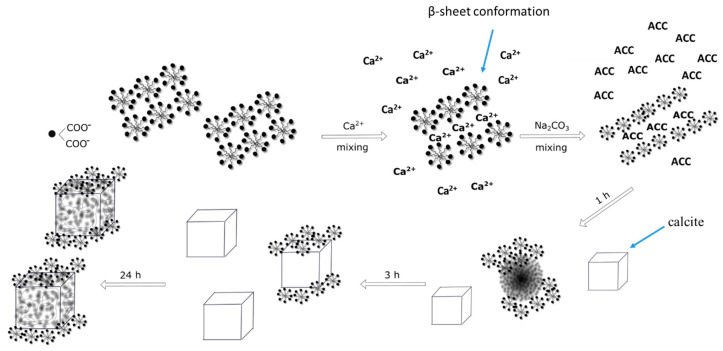
Mechanism of mesoporous calcite formation.

Surfactin is a lipopeptide biosurfactant composed of a cyclic ring of amino acids linked to hydroxylic fatty acids by a lactone bond [41,42]. The cyclic peptide part contains two hydrophilic residues of Glu and Asp acid because of free groups of COO^−^ at a pH above pK_a_ = 5.8 [43,44]. These two negatively charged groups in the surfactin molecule are considered to form complexes with Ca^2+^ ions at pH 8 and enhanced the intermicellar aggregates formation in β-sheet conformation, as discussed in detail in the literature [45,46]. This can slightly reduce the concentration of free Ca^2+^ during the nucleation stage (Figure 8), because in the system an excess of calcium and carbonate ions compared to surfactin molecules was used. Moreover, the life span of this bond is short, and when carbonate ions are added, the released and free Ca^2+^ ions interact with CO_3_^2−^ ions [16] and form hydrated nuclei and vaterite-calcite mixture in the subsequent step. The free surfactin molecules can be adsorbed onto the selected crystals phase. It is known from the literature that during crystallization, vaterite particles may have a positive charge on the surface [28]. Surfactin molecules can adsorb on the vaterite surface via electrostatic interactions as well as via hydrogen bonds between protonated amino groups and oxygen in the carbonate group on the crystal surface [47,48]. The presence of surfactin molecules on the solid/liquid interface changed the solubility of vaterite [18]. As a result, further conversion of crystals into calcite slowed down (Table 5), followed by gradual changes in morphology (Figure 6), crystallite size (Figure 7C) and microstrain (Figure 7D). Additionally, changes in the *T*(%) value (Figure 1A) prove that the small particles are dispersed in the system. This inhibiting effect of surfactin intensifies as the biomolecule concentration increases. More vaterite remained in the system after 24 h of ageing when more biosurfactant was added (Table 2). A similar mechanism was proposed by Wu et al. (2018) [49], where citrate anions were used as a modifying agent. However, in this case, the surface morphology of calcite was significantly deformed (Figure 3D,E and Figure 6F). Some slight cavities appeared on the surface of calcite crystals obtained in the presence of 10 ppm surfactin after 24 h of ageing. Moreover, the edges of calcite particles obtained after 24 h were blunted, and micelles embedded in crystals were observed (Figure S5.), which was also confirmed by the lattice parameter values (Table 2), where positive distortion was observed. Additionally, TGA and QMS analyses (Figure 5B) prove the occlusion of the surfactin molecules. Our results confirm the hypothesis that the deformation of the crystals results from the existence of micelles in the reaction medium. Prolonging the time of reaction up to 24 h, the percentage of vaterite in the sample decreased and more calcite is formed (Table 5) due to the dissolution of the metastable phase. The released inorganic ions as well as the aggregates of micelles may accumulate (according to the diffusion gradient) near the surface of large crystals formed earlier (Figure 8). The ions interact with the external functional group of the surfactin, and the new calcite grains are crystallized. The clusters formed are attached to the calcite surface, and finally, mesoporous calcite crystals with a small diameter and larger specific surface area are produced after 24 h of ageing. The mechanism of calcite formation in the presence of surfactin is similar to that which occurs in nature [50]. The presence of micelles at the surface of crystals formed can block further growth due to the decrease in the zeta potential value of crystals formed. This has reduced crystal aggregation via electrostatic repulsion between the crystals. This was confirmed by zeta potential and *T* changes (Figure 1A,B), SEM images (Figure 6), crystallite size (Figure 7A), and the size distribution of CaCO_3_ particles (Figure S1). The higher the concentration of biosurfactant in the solution, the more micelles were incorporated on the crystal surface, as confirmed by SEM images (Figure 3), the zeta potential values (Figure 2) and TGA (Figure 5B). Additionally, the size of pores (cavities) on the surface is close to the size of a single micelle of biosurfactant, ca. 10 nm [51]. It is characteristic that the surface roughness increases with time (Figure 6). Additionally, the size of crystallites in the sample with surfactin after 24 h of ageing is significantly lower than in the control sample (Figure 7A). This results from oval and spherical depletions on the calcite crystal surface [52]. On the calcite surface, the edges are more rounded and truncated, indicating the presence of micelles on the edges of the crystals. The surface of calcite was less deformed when there was more vaterite in the system. Since calcite has a negative zeta potential value (Figure 1B), negatively charged micelles prefer to adsorb on a positively charged vaterite surface. This explains why more vaterite than calcite was produced in the presence of surfactin molecules. Thus, as long as vaterite particles are present in the system, the calcite surface will not be significantly distorted, as shown in Figure 6. 

## 3. Materials and Methods

Calcium chloride from Fluka (Bucharest, Romania) and sodium carbonate (p.a.) from Avantor Performance Materials Poland S.A. (Gliwice, Poland) were used as reactants. Surfactin (purity 91%) was purchased from Lipofabrik (France). The procedure of calcium carbonate microstructure synthesis was described in Bastrzyk et al. (2019) [16]. First, the calcium chloride solution was conditioned with surfactin at various concentrations at pH 8. Then, the CaCO_3_ crystals were obtained by mixing two 0.01 M equimolar solutions of CaCl_2_ (without and with the biosurfactant) and Na_2_CO_3_ at constant stirring intensity using a magnetic stirrer. After adding Na_2_CO_3_, the pH of the reaction medium was increased to 10.5. The reaction times were 30 min, 1 h, 3 h, and 24 h. After the mixing time, the precipitate was separated, dried and characterized. The concentrations of biosurfactant were 5, 10 and 20 ppm. The control sample was synthesized without the biosurfactant. All experiments were carried out at room temperature (25 °C), and three separate experiments were performed.

The precipitate was characterized after drying using scanning electron microscopy (SEM), Brunauer-Emmett-Teller (BET) analysis and thermogravimetry (TG) coupled with gas mass spectrometry (QMS) analysis. The particle size distribution and zeta potential value of CaCO_3_ were also measured. All procedures for characterizing the CaCO_3_ particles are included in the Supplementary Materials (please see Section S1.1).

All reagents were used without further purification. Water was provided from a WG-LHP purification system (WIGO, Poland) with a conductivity of 0.055 μScm^−1^.

## 4. Conclusions

Based on the research carried out, it can be concluded that surfactin can effectively affect the properties of precipitated calcium carbonate. Light transmittance value changes confirmed the hypothesis that biosurfactant molecules delayed the growth of calcium carbonate crystals. The presence of biosurfactant in the reaction system significantly changed the precipitate morphology, especially for calcite. The structure of the crystals was deformed, and after 24 h of reaction, mesoporous calcium carbonate was obtained at a low biosurfactant concentration (10 ppm). The specific surface area of the porous calcite was 10 times larger than that of the control sample without surfactin. The porous structure of calcium carbonate results from the adsorption and occlusion of micelles in the crystals during growth. The pore size was similar to the size of a single micelle, indicating the adsorption of micelles on calcite crystals during crystallization, which was proven by zeta potential measurements, lattice parameter calculations and TGA. The incorporation of micelles did not change the thermal properties of the particles. Moreover, the biomolecules can be removed from the crystals by heating at temperatures below 200 °C. These studies provided valuable information on the formation of inorganic matrices with unique morphologies and surface properties, which can be of great benefit for biomedical and environmental applications.

In addition, this is the first report on the study of calcite growth over time in a micelle system of an environmentally friendly surfactant.

## Figures and Tables

**Table 1:** Median diameter (*d_50_*), lower (*d_10_*) and upper (*d_90_*) deciles of calcium carbonate particles obtained in the presence of biosurfactant after 24 h of ageing. (Standard deviation for *d* values was lower than 1 µ).

Surfactin Concentration [ppm]	*d_10_* [µm]	*d_50_* [µm]	*d_90_*[µm]
0	12.5	24.4	44.6
5	8.4	15.4	26.0
10	6.3	12.4	21.6
20	5.0	10.6	19.6

**Table 2:** Phase composition and the elemental cell parameters of the precipitated calcium carbonate calculated from the diffraction patterns. (Standard deviation for calcite or vaterite content was lower than 2%. Standard deviation for calcite cell parameters a, c and *V* were 0.003–0.008%, 0.001−0.006%, 0.006−0.013%, respectively. Standard deviation for vaterite cell parameters a, c and *V* were 0.011–0.013%, 0.025–0.029%, 0.23–0.34%, respectively).

Surfactin Concentration [ppm]	Calcite		Vaterite	
	Content [%]	Structural Parameters	Content [%]	Structural Parameters
0	100	*a* = *b* = 4.9900(2) Å	-	-
		*c* = 17.0550(7) Å		
		*α* = *β* = 90°, *γ* = 120°		
		*V* = 367.776 (Å)^3^		
5	100	*a* = *b* = 4.9923(3) Å	-	-
		*c* = 17.0537(14) Å		
		*α* = *β* = 90°, *γ* = 120°		
		*V* = 368.087 (Å)^3^		
10	98.6	*a* = *b* = 4.9927(1) Å	1.4	*a* = *b* = 4.1268(4) Å
		*c* = 17.0608(6) Å		*c* = 8.4791(20) Å
		*α* = *β* = 90°, *γ* = 120°		*α* = *β* = 90°, *γ* = 120°
		*V* = 368.299 (Å)^3^		*V* = 125.057 (Å)^3^
20	85.1	*a* = *b* = 4.9926(2) Å	14.9	*a* = *b* = 4.1299(5) Å
		*c* = 17.0619(9) Å		*c* = 8.4699(25) Å
		*α* = *β* = 90°, *γ* = 120°		*α* = *β* = 90°, *γ* = 120°
		*V* = 368.308 (Å)^3^		*V* = 125.109 (Å)^3^

**Table 3:** The characteristic surface parameters of CaCO_3_ precipitate.

Surfactin Concentration [ppm]	SSA [m^2^g^−1^]	Average Pores Volume [cm^3^g^−1^]	Average Pores Diameter [nm]
0	0.18 ± 0.05	-	-
10	1.67 ± 0.1	0.00513 ± 0.0002	8.7 ± 1.0
20	4.87 ± 0.09	0.0123 ± 0.001	11.7 ± 1.2

**Table 4:** Thermogravimetry results of the samples (Δ*_m_*–weight loss, *T*–maximum on the DTG curve, onset-temperature- of main degradation start).

Surfactin Concentration [ppm]	∆*m_1_* (25–400 °C) [%]	∆*m_2_* (400–566 °C) [%]	*T_1_* [°C]	*T_2_* [°C]	∆*m_total_* [%]
0	0.71		-	717	43.69
20	0.95	0.95	510	724	44.71

**Table 5:** Phase composition of precipitated calcium carbonate calculated from the diffraction patterns. (Standard deviation for calcite/vaterite content was lower than 1%). Surfactin concentration was 10 ppm. pH was 8.

Time [hours]	Calcite [%]	Vaterite [%]
0.5	59.6	40.5
1	67.8	32.2
3	90.5	9.5
24	98.6	1.4

## Supplementary Materials

Supplementary materials can be found at https://www.mdpi.com/1422-0067/21/15/5526/s1.
