# Supplementary material for: Surfactin as a Green Agent Controlling the Growth of Porous Calcite Microstructures
Source: Int J Mol Sci. 2020 Aug 1;21(15):5526. doi: 10.3390/ijms21155526 (PMC7432438; doi:10.3390/ijms21155526)
Supplement: Supplementary file 1 [file ijms-21-05526-s001.pdf]

Supplementary Material

Surfactin as a Green Agent Controlling the Growth of Porous Calcite Microstructures

Anna Bastrzyk [1,*], Marta Fiedot-Toboła [2,*], Halina Maniak [3], Izabela Polowczyk [1] and Grażyna Płaza [4]

[1] Department of Process Engineering and Technology of Polymer and Carbon Materials, Faculty of Chemistry, Wrocław University of Science and Technology, Wybrzeże Wyspiańskiego 27, 50-370 Wrocław, Poland; anna.bastrzyk@pwr.edu.pl(A.B.); izabela.polowczyk@pwr.edu.pl(I.P.)

[2] Łukasiewicz Research Network-PORT Polish Center for Technology Development, Stabłowicka 147, 54-066 Wrocław, Poland; marta.fiedot-tobola@port.lukasiewicz.gov.pl

[3] Department of Micro, Nano and Bioprocess Engineering, Faculty of Chemistry, Wrocław University of Science and Technology, Wybrzeże Wyspiańskiego 27, 50-370 Wrocław, Poland; halina.maniak@pwr.edu.pl

[4] Department of Environmental Microbiology, Institute for Ecology of Industrial Areas, Kossutha 6, 40-844 Katowice, Poland; g.plaza@ietu.pl

* Correspondence: anna.bastrzyk@pwr.edu.pl (A.B.); marta.fiedot-tobola@port.lukasiewicz.gov.pl (M.F.T.); Tel.: +48-71-320-32-39 (A.B.); +48-71-734-71-54 (M.F.T.)

S.1. Experimental section

S.1.1. Calcium carbonate crystals characterization

Precipitate morphology was investigated by scanning electron microscopy using an SEM/Xe-PFIB Microscope FEI Helios PFIB (USA). The accelerating electron voltage was 2 kV, and the working distance was 10 mm.

The crystal polymorph was measured by X-ray diffraction using a D8 ADVANCE diffractometer (Bruker, Massachusetts, USA) with Co radiation. Measurements were carried out by a step recording method with a shift $\Delta 2\Theta = 0.02°$ in the angle range 10–60° 2 Θ.

For BET measurement, samples were degassed in a Smart VacPrep preparatory station (Micromeritics Instrument Corp., Norcross, U.S.A.) at 100°C for 12 hours under vacuum conditions. The adsorption and desorption isotherms were determined for the mesoporous materials under liquid nitrogen (99.9999%). Pure helium was used to measure pore size. The analyses were performed on 3Flex analyzer (Micromeritics Instrument Corp., Norcross, U.S.A.). Using the Brunauer, Emmett and Teller (BET) method, the surface area values were determined. Next, the Barrett–Joyner–Halenda (BJH) method was used to determine pore volumes. Finally, the average pore size was determined based on density functional theory (DFT).

Thermal properties of the materials were investigated using thermogravimetry (TG). Analysis were performed in the temperature range 25–850°C with the heating rates 5, 10, 15, or 20 $K{\cdot}min^{-1}$ in nitrogen atmosphere (30 $ml{\cdot}min^{-1}$). T using thermobalance TGA2 (Mettler Toledo, Greifensee, Switzerland) were used. E_a value changes as a function of material conversion were determined using model-free kinetics invented by Vyazovkina. To define the energy effect and gas produced during the samples heating, the differential scanning calorimeter (DSC) coupled with mass spectroscopy (QMS) (STA 449 F1 Jupiter; QMS Aeolos, Netzsch, Selb, Germany) was used.

The size distribution of crystals was determined using a Mastersizer 2000 laser diffractometer, equipped with HydroMu dispersion unit (Malvern, UK). A dedicated program also calculates a number of diameters, most of which are used to characterize a population of particles, the so-called median diameter, d_{50}, and the first and the ninth decile (d_{10} and d_{90}). These diameters correspond to a diameter that does not exceed 50, 10 and 90% of the particle population, respectively.

The zeta potential (ξ) values of particles were measured using a Zetasizer 2000 apparatus (Malvern, UK). In this test, 0.01g of dried calcium carbonate powder was weighted into a glass beaker and then 20 ml of 1mM NaCl solution was added. The pH of the suspension was adjusted to 7, 8, 9, and 10 using 1 mM HCl or NaOH solution. The suspension was introduced into the measuring cell of the apparatus and the zeta potential value of the particles was measured. Each experiment was conducted twice. The average value of ξ was taken from five sets of measurements.

The stability of the suspensions during calcium carbonate synthesis was investigated using a Turbiscan LabEXPERT instrument (Formulaction, L'Union, France). The apparatus can detect and measure even small changes in the suspension behavior by means of transmission (T) and backscattering (BS) light collection. The light source is an electroluminescent diode (λ_{air} = 880 nm). Two synchronous optical sensors gather the light transmitted through the sample and the light backscattered by the sample. In the Turbiscan Lab, the optical reading head scans a sample every 40 µm to acquire the transmission and backscattering data. Transmission is used to analyze transparent to turbid dispersions, and backscattering is used to analyze opaque dispersions. The samples were prepared in a special glass cell. The procedure was analogous to that applied in the synthesis method, except that the final volume was 20 ml and the mixing time was 5 minutes. The biosurfactant concentration in the tested sample was 10 ppm. The control sample without surfactin was also prepared. A glass cell containing the sample was then placed in the Turbiscan apparatus and changes in T and BS were monitored.

S.2. Results and Discussion

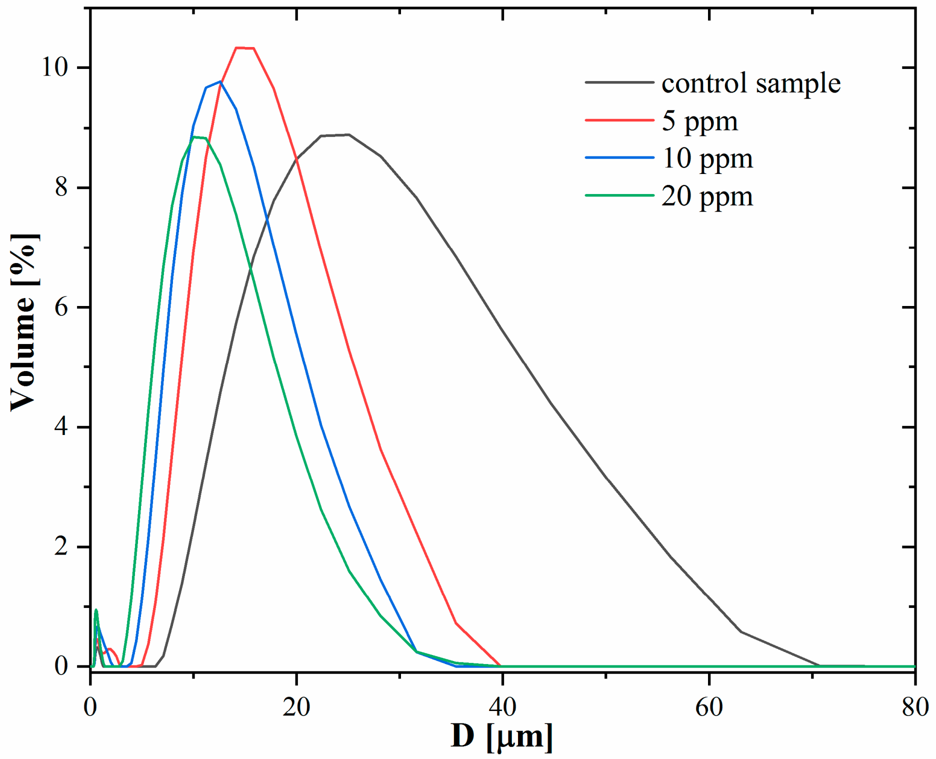

Figure S1. Particle size distribution of calcium carbonate precipitated at various surfactin concentrations after 24 hours of ageing. The concentration of $CaCl_2$ and Na_2CO_3 was 5 mM.

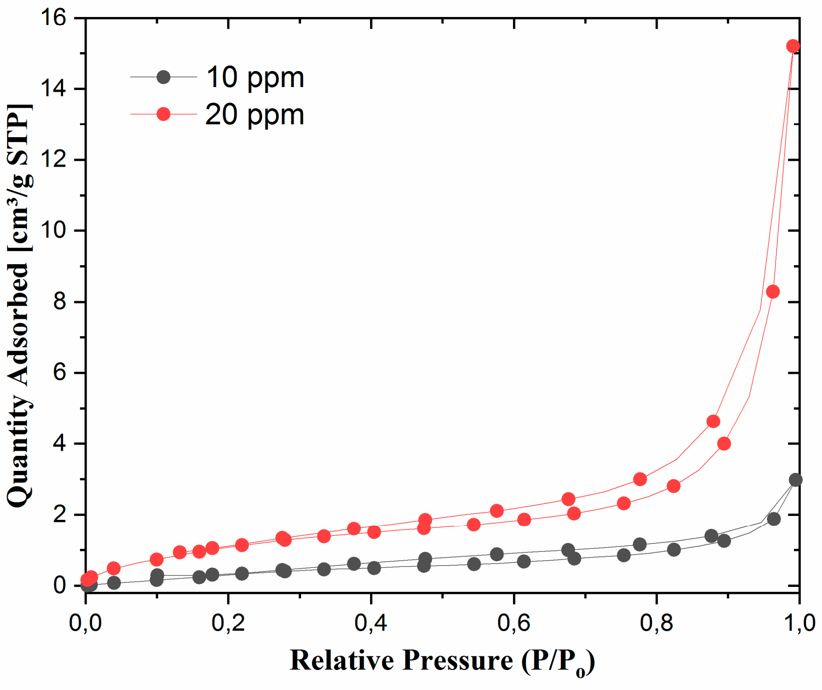

Figure S2. Adsorption and desorption isotherms of nitrogen on calcium carbonate precipitated in the presence of surfactin at pH 8.

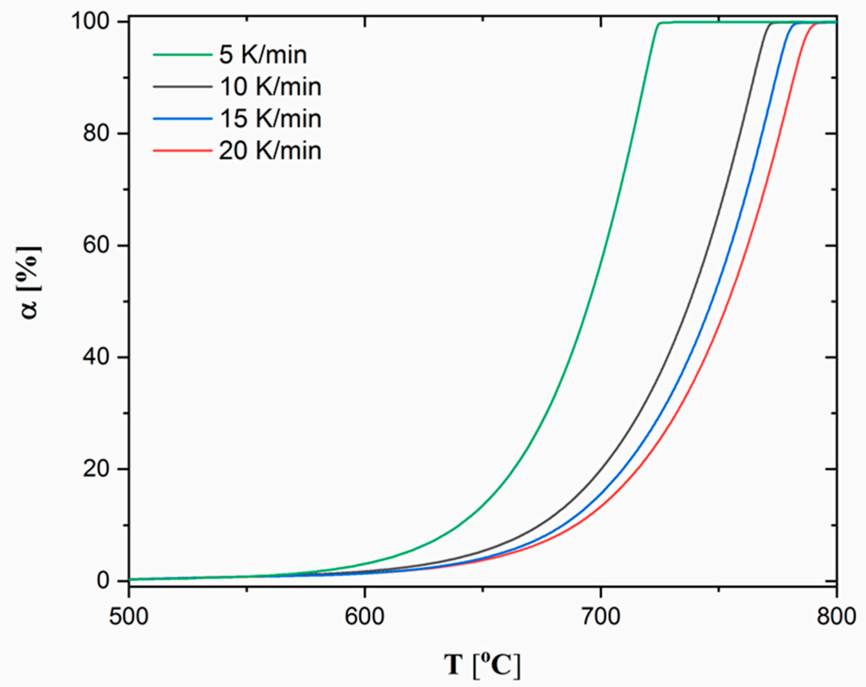

Figure S3. Conversion curve with different heating rates.

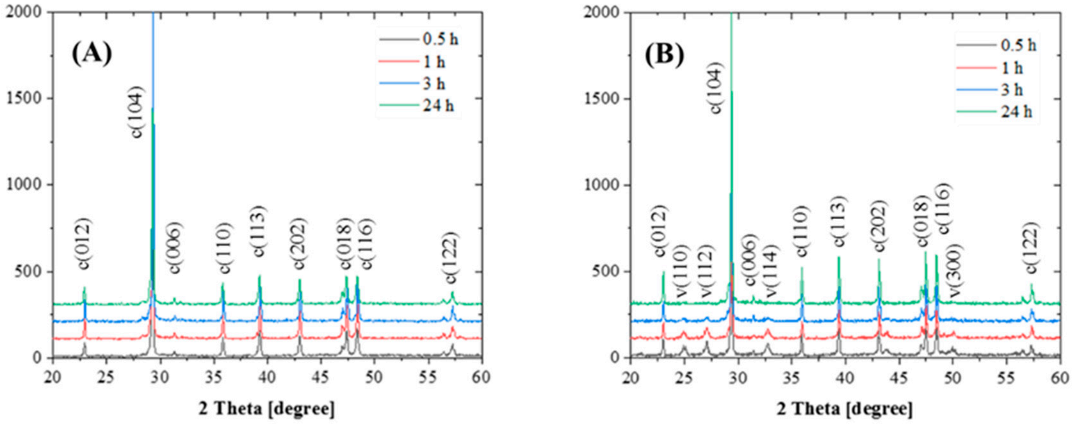

Figure S4. XRD patterns of calcium carbonate precipitation at various of time ageing. **A**: control sample, **B**: 10 ppm of surfactin. The concentration of $CaCl_2$ and Na_2CO_3 was 5mM.

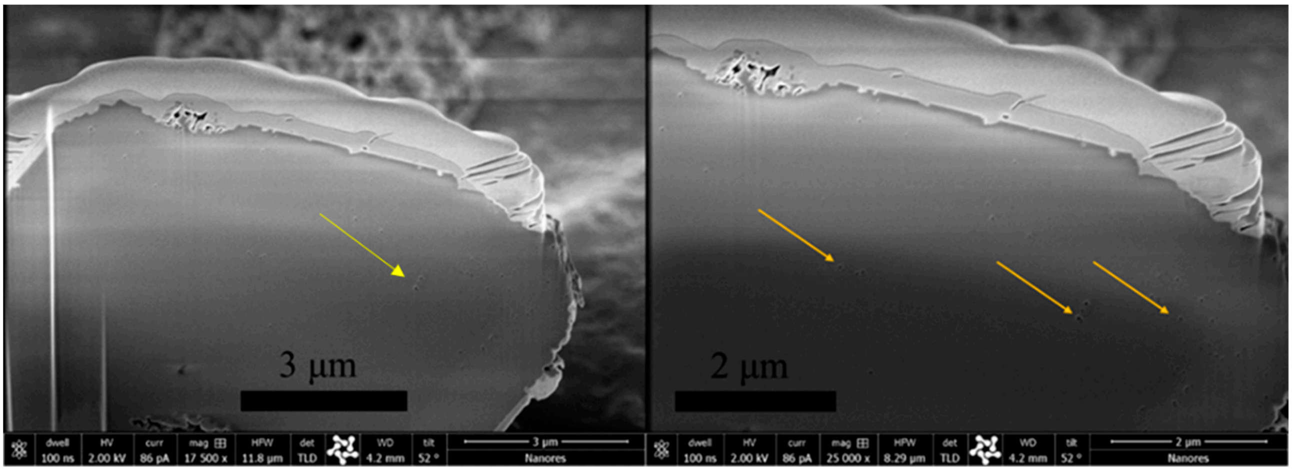

Figure S5. Etched calcite crystals obtained in the presence of 10 ppm of surfactin (pores marked with yellow arrow).
